# Molecular Phylogeny of Mobatviruses (*Hantaviridae*) in Myanmar and Vietnam

**DOI:** 10.3390/v11030228

**Published:** 2019-03-07

**Authors:** Satoru Arai, Fuka Kikuchi, Saw Bawm, Nguyễn Trường Sơn, Kyaw San Lin, Vương Tân Tú, Keita Aoki, Kimiyuki Tsuchiya, Keiko Tanaka-Taya, Shigeru Morikawa, Kazunori Oishi, Richard Yanagihara

**Affiliations:** 1 Infectious Disease Surveillance Center, National Institute of Infectious Diseases, Tokyo 162-8640, Japan; arais@nih.go.jp (S.A.); fkikuchi@niid.go.jp (F.K.); 1718501@ed.tus.ac.jp (K.A.); ktaya@nih.go.jp (K.T.-T.); oishik@nih.go.jp (K.O.); 2 Department of Chemistry, Faculty of Science, Tokyo University of Science, Tokyo 162-8601, Japan; 3 Department of Pharmacology and Parasitology, University of Veterinary Science, Yezin, Nay Pyi Taw 15013, Myanmar; bestshadow@gmail.com; 4 Institute of Ecology and Biological Resources, Vietnam Academy of Science and Technology, Hanoi, Vietnam; ntson@iebr.vast.vn (N.T.S.); vttu@iebr.ac.vn (V.T.T.); 5 Graduate University of Science and Technology, Vietnam Academy of Science and Technology, Hanoi, Vietnam; 6 Department of Aquaculture and Aquatic Disease, University of Veterinary Science, Yezin, Nay Pyi Taw 15013, Myanmar; kyawsanlinuvs@gmail.com; 7 Department of Liberal Arts, Faculty of Science, Tokyo University of Science, Tokyo 162-8601, Japan; 8 Laboratory of Bioresources, Applied Biology Co., Ltd., Tokyo 107-0062, Japan; tsuchiya@seibutsu.co.jp; 9 Department of Veterinary Science, National Institute of Infectious Diseases, Tokyo 162-8640, Japan; morikawa@nih.go.jp; 10 Pacific Center for Emerging Infectious Diseases Research, John A. Burns School of Medicine, University of Hawaii at Manoa, Honolulu, HI 96813, USA

**Keywords:** *Hantaviridae*, *Mobatvirus*, phylogeny

## Abstract

The discovery of highly divergent lineages of hantaviruses (family *Hantaviridae*) in shrews, moles, and bats of multiple species raises the possibility that non-rodent hosts may have played a significant role in their evolutionary history. To further investigate this prospect, total RNA was extracted from RNAlater^®^-preserved lung tissues of 277 bats (representing five families, 14 genera and 40 species), captured in Myanmar and Vietnam during 2013–2016. Hantavirus RNA was detected in two of 15 black-bearded tomb bats (*Taphozous melanopogon*) and two of 26 Pomona roundleaf bats (*Hipposideros pomona*) in Myanmar, and in three of six ashy leaf-nosed bats (*Hipposideros cineraceus*) in Vietnam. Pair-wise alignment and comparison of coding regions of the S, M, and L segments of hantaviruses from *Taphozous* and *Hipposideros* bats revealed high nucleotide and amino acid sequence similarities to prototype Láibīn virus (LAIV) and Xuân Sơn virus (XSV), respectively. Phylogenetic analyses, generated by maximum-likelihood and Bayesian methods, showed a geographic clustering of LAIV strains from China and Myanmar, but not of XSV strains from China and Vietnam. These findings confirm that the black-bearded tomb bat is the natural reservoir of LAIV, and that more than one species of *Hipposideros* bats can host XSV.

## 1. Introduction

Based on the phylogenetic analysis of the full-length S and M segments, members of the genus *Hantavirus* in the former family *Bunyaviridae* have been recently reclassified into a new family, designated *Hantaviridae*, of the order *Bunyavirales* [1,2]. In this revised taxonomic classification, hantaviruses have been assigned to four newly defined genera: *Loanvirus*, *Mobatvirus*, *Orthohantavirus*, and *Thottimvirus* [2]. All hantaviruses harbored by rodents, including those associated with hemorrhagic fever with renal syndrome and hantavirus pulmonary syndrome, belong to the genus *Orthohantavirus*, which also comprises nearly all of the genetically distinct hantaviruses recently detected in shrews and moles of multiple species (order Eulipotyphla, family Soricidae and Talpidae) from widely separated geographic regions in Asia, Europe, Africa and/or North America [2,3].

The genus *Thottimvirus* includes Thottapalayam virus (TPMV) in the Asian house shrew (*Suncus murinus*) [4,5,6], and Imjin virus (MJNV) in the Ussuri white-toothed shrew (*Crocidura lasiura*) [7]. Although sequence data are incomplete, Uluguru virus (ULUV) in the geata mouse shrew (*Myosorex geata*) [8] and Kilimanjaro virus (KMJV) in the Kilimanjaro mouse shrew (*Myosorex zinki*) [8] from Tanzania are also likely members of the genus *Thottimvirus* [3].

By contrast, hantaviruses hosted by bats (order Chiropera, suborder Yangochiroptera, and Yinpterochiroptera) have been assigned to the *Loanvirus* and *Mobatvirus* genera [2,3]. Members of the genus *Loanvirus* include Lóngquán virus (LQUV) in the intermediate horseshoe bat (*Rhinolophus affinis*), Formosan lesser horseshoe bat (*Rhinolophus monoceros*) and Chinese rufous horseshoe bat (*Rhinolophus sinicus*) [9] from China, and Brno virus (BRNV) in the common noctule (*Nyctalus noctula*) [10] from the Czech Republic. Other likely members include the Magboi virus (MGBV) in the hairy slit-faced bat (*Nycteris hispida*) [11] from Sierra Leone, Mouyassué virus (MOYV) in the banana pipistrelle (*Neoromicia nanus*) [12,13] from Côte d’Ivoire and in the cape serotine (*Neoromicia capensis*) [14] from Ethiopia, and Huángpí virus (HUPV) in the Japanese house bat (*Pipistrellus abramus*) [9] from China.

Members of the genus *Mobatvirus* include Láibīn virus (LAIV) in the black-bearded tomb bat (*Taphozous melanopogon*) [15] from China, and Quezon virus (QZNV) in the Geoffroy’s rousette (*Rousettus amplexicaudatus*) [16] from the Philippines. Other bat-borne hantaviruses which likely belong to the genus *Mobatvirus* include Xuân Sơn (XSV) virus in the Pomona roundleaf bat (*Hipposideros pomona*) [13,17] from Vietnam, Makokou virus (MAKV) in the Noack’s roundleaf bat (*Hipposideros ruber*) [18] from Gabon, and Dakrong virus (DKGV) in the Stoliczka’s Asian trident bat (*Aselliscus stoliczkanus*) [19] from Vietnam. Nova virus (NVAV), a highly divergent hantavirus harbored by the European mole (*Talpa europaea*) in Hungary [20], France [21], Poland [22], and Belgium [23], is the only mobatvirus not hosted by a bat species. 

The realization that bats of multiple species harbor loanviruses and mobatviruses that are more genetically diverse than orthohantaviruses hosted by rodents and shrews suggests that ancestral bats may have served as the primordial hosts of hantaviruses [3,12,24,25]. Of the 10 bat-borne hantaviruses reported to date, full-length genomes are available for BRNV, DKGV, LAIV, QZNV, and XSV. That said, data about the genetic diversity and phylogeography of loanviruses and mobatviruses are largely unavailable. To address this gap in knowledge, we detected and analyzed hantavirus genomes in lung tissues from bats (representing five families, 14 genera and 40 species), captured in Myanmar and Vietnam. 

## 2. Materials and Methods

### 2.1. Samples

RNAlater^®^-preserved lung tissues from 277 bats, of which 121 were captured in Myanmar and 156 in Vietnam during 2013–2016 (Table 1), were analyzed for hantavirus RNA by nested RT-PCR, using previously employed oligonucleotide primers [12,13,16,17,19]. Tested bats were from five families (Emballonuridae, Hipposideridae, Pteropodidae, Rhinolophidae, and Vespertilionidae), 14 genera, and 40 species.

### 2.2. Genome Detection and Sequencing

Total RNA was extracted from lung tissues, using the MagDEA RNA 100 Kit (Precision System Science, Matsudo, Japan), and complementary DNA (cDNA) was synthesized, using the PrimeScript II First strand cDNA Synthesis Kit (Takara Bio, Inc., Otsu, Japan) with oligonucleotide primer (OSM55F, 5′–TAGTAGTAGACTCC–3′), designed from the conserved 5′-ends of the S-, M-, and L-segments of hantaviruses [20]. Oligonucleotide primers used to amplify S-, M-, and L-genomic segments of bat-borne hantaviruses are listed on Table 1. First- and second-round PCR reactions were performed in 20 μL reaction mixtures, containing 250 μM dNTPs, 2.5 mM MgCl_2_, 1 U of Takara LA Taq polymerase Host Start version (Takara Bio, Inc., Ohtsu, Japan), and 0.25 μM of each primer [26]. Initial denaturation at 94 °C for 2 min was followed by two cycles each of denaturation at 94 °C for 30 s, two-degree step-down annealing from 46 °C to 38 °C for 40 s, and elongation at 72 °C for 1 min, then 30 cycles of denaturation at 94 °C for 30 s, annealing at 42 °C for 40 s, and elongation at 72 °C for 1 min, in a Veriti thermal cycler (Applied Biosystems, Foster City, CA, USA) [16,26,27,28,29,30]. PCR products, treated with ExoSAP-IT (Thermo Fisher Science, Waltham, MA, USA) according to the manufacturer’s instruction, were sequenced directly, using an ABI 3730xl DNA Analyzer (Applied Biosystems) [24,25].

### 2.3. Phylogenetic Analysis

Maximum-likelihood and Bayesian methods, implemented in MrBayes 3.1 [31], under the best-fit GTR + I + Γ model of evolution [32] and jModelTest [33], were used to generate phylogenetic trees. Two replicate Bayesian Metropolis–Hastings Markov chain Monte Carlo runs, each consisting of six chains of 10 million generations sampled every 100 generations with a burn-in of 25,000 (25%), resulted in 150,000 trees overall. The S, M, and L segments were treated separately in phylogenetic analyses. Topologies were evaluated by a bootstrap analysis of 1000 iterations, and posterior node probabilities were based on two million generations, and sample sizes were estimated to be over 100 (implemented in MrBayes). Parameters were re-estimated during successive rounds of maximum-likelihood heuristic searches using the tree bisection reconnection and subtree-pruning–regrafting algorithms implemented in PAUP*.

### 2.4. Host Identification and Phylogeny

Total DNA was extracted from the lung tissues of bats, using MagDEA DNA 200 Kit (Precision System Science, Matsudo, Japan), and PCR amplification of the cytochrome *b* (Cyt *b*) and cytochrome oxidase I (COI) genes of selected bats was performed using primer sets: Cy-14724F (5′–GACYARTRRCATGAAAAAYCAYCGTTGT–3′)/Cy-15909R (5′–CYYCWTYIYTGGTTTACAAGAC YAG–3′) [34] and KOD multi-enzyme (Toyobo, Osaka, Japan), and MammMt-5533F (5′–CYCTGTSYTTRRATTTACAGTYYAA–3′)/MammMt-7159R (5′–GRGGTTCRAWWCCTYCCTYTCT T–3′) and Phusion enzyme (New England Biolabs, Ipswitch, MA, USA), respectively. Initial denaturation was at 95 °C for 2 min, and PCR was performed, using two cycles each of denaturation at 95 °C for 15 s, step-down annealing from 60 °C to 50 °C every two degrees for 30 s, and elongation at 68 °C for 1 min 30 s, then 30 cycles of denaturation at 95 °C for 15 s, annealing at 55 °C for 30 s, and elongation at 68 °C for 1 min 30 s, in a Veriti thermal cycler. PCR products were purified by a Mobispin S-400 (Molecular Biotechnology, Lotzzestrasse, Germany), and were sequenced directly. The newly generated sequences were then edited, assembled using ATGC bundled in Genetyx (v. 13) (Genetyx, Shibuya, Tokyo, Japan), and deposited in GenBank under the accession numbers MK410312–MK410432, MK430027–MK430032, and MK462230–MK462234 (Table S4).

To explore the phylogenetic relationships of the hosts of mobatviruses, out-group species were selected, including Macroscelidea (genus *Elephantulus*), as well as small mammals of the orders Eulipotyphla (families Soricidae and Talpidae) and Rodentia (families Muridae and Cricetidae), which are known to serve as reservoir hosts of hantaviruses (Table S4). Phylogenetic relationships were inferred from sequencing analysis of the entire 1140-nucleotide Cyt *b* (54 taxa), and the 1545-nucleotide COI (53 taxa) genes of mitochondrial DNA, using respective models of sequence evolution (GTR+I+Γ for Cyt *b* and HYK+I+Γ for COI), selected with jModelTest v2.1.6 [35]. Posterior probabilities were calculated by using two replicate Markov chain Monte Carlo runs, consisting of six chains of 10 million generations, each sampled every 100 generations, with a burn-in of 25,000 (25%).

## 3. Results and Discussion

### 3.1. Virus Detection

**Figure 1:**
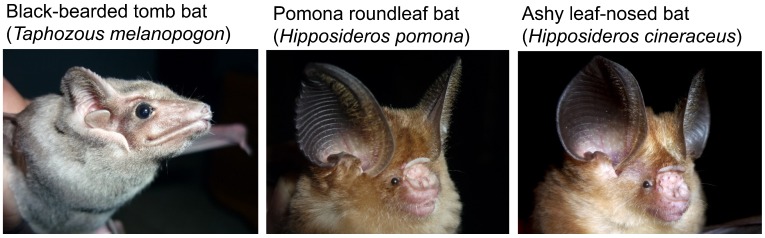
Insectivorous bats harboring mobatviruses in Myanmar and Vietnam. The black-bearded tomb bat (*Taphozous melanopogon*) (family Emballonuridae) hosts Láibīn virus, and the Pomona roundleaf bat (*Hipposideros pomona*) and ashy leaf-nosed bat (*Hipposideros cineraceus*) (family Hipposideridae) hosts Xuân Sơn virus.

**Figure 2:**
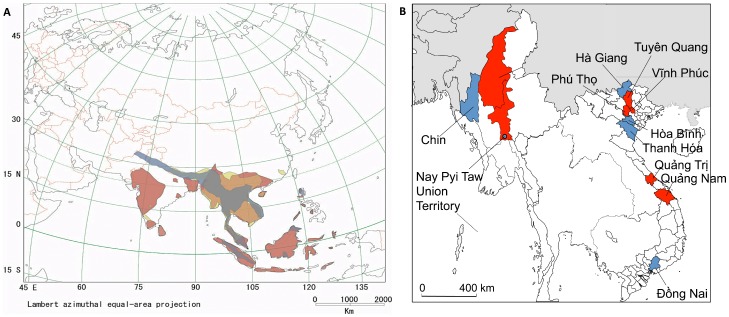
(**A**) Geographic distribution of the black-bearded tomb bat (*Taphozous melanopogon*) (suborder Yangochiroptera, family Emballonuridae) (colored rust), Pomona roundleaf bat (*Hipposideros pomona*) (suborder Yinpterochiroptera, family Hipposideridae) (maize), and ashy leaf-nosed bat (*Hipposideros cineraceus*) (suborder Yinpterochiroptera, family Hipposideridae) (grey). Areas of overlap between the bat species are colored brown. (**B**) Bats were captured in seven provinces in Vietnam, and three districts in Myanmar (colored red and blue). Capture sites in Vietnam and Myanmar yielding bats infected with Láibīn virus and Xuân Sơn virus are shown in red.

LAIV RNA was detected by nested RT-PCR in two of 15 black-bearded tomb bats captured in Shwe Ba Hill Cave (22.05680555 N, 94.97880555 E) in the Sagaing Region of Myanmar (Table 2 and Figure 1). In addition, XSV RNA was found in one of 25 Pomona roundleaf bats captured in a nearby forest (22.056788 N, 94.978702 E) in the Sagaing Region, and in a single Pomona roundleaf bat trapped near a hotel (19.864542 N, 96.158342 E) in Nay Pyi Taw Union Territory in Myanmar, as well as in three of six ashy leaf-nosed bats (*Hipposideros cineraceus*) captured in Vietnam: one XSV strain each in Bắc Hướng Hóa Nature Reserve (16.8891 N 106.5705 E) in Hướng Hóa District, Quảng Trị Province, in Xuân Sơn National Park (21.123103 N, 104.960002 E) in Tân Sơn District, Phú Thọ Province, and in Me Linh Station for Biodiversity (21.123103 N, 104.960002 E) in Phúc Yên District, Vĩnh Phúc Province (Table 2 and Figure 2).

The overly simplistic view that each genetically distinct hantavirus is harbored by a single reservoir host species (with which it co-evolved) is no longer tenable, as evidenced by multiple examples of host sharing, or the hosting of hantaviruses of the same species by more than one closely-related reservoir species [3,24,25]. The demonstration that XSV is harbored by two species of *Hipposideros* bats serves as another example of host sharing, similar to earlier reported examples, such as MOYV in *Neoromicia nanus* [12,13] and *Neoromicia capensis* [14], and LQUV in *Rhinolophus affinis*, *Rhinolophus monoceros* and *Rhinolophus sinicus* [9]. Multiple other examples of host sharing have also been reported for orthohantaviruses harbored by rodents, shrews and moles [3].

Despite employing oligonucleotide primers and PCR cycling conditions used previously to detect MOYV [12,13], XSV [13,17] and QZNV [16], repeated attempts failed to uncover hantavirus RNA in all other bat samples, including lung tissues from bat species previously reported to harbor loanviruses and mobatviruses. That is, tissues from 10 Japanese house bats, 15 intermediate horseshoe bats, seven Chinese rufous horseshoe bats, and four Stoliczka’s Asian trident bats were negative. The reasons for the overall low success rates of detecting mobatvirus or loanvirus RNA in bat tissues may be the highly divergent nature of their genomes, the very focal or localized nature of infection in bats, the small sample sizes, the suboptimal tissue preservation with degraded RNA, and the low virus load. Another possibility may be that bats are less susceptible to mobatvirus or loanvirus infection, or that bats have immune mechanisms to curtail viral replication and/or persistence.

### 3.2. Sequence Analysis

Pair-wise alignments of the partial and full-length sequences of S, M, and L segments of mobatviruses from *Taphozous* and *Hipposideros* bats were compared with prototype LAIV and XSV strains, as well as other LAIV and XSV strains available in GenBank (Table 3). The near-full-length S (1774 nucleotides) and M (3881 nucleotides) segment sequences, and the full-length L (6531 nucleotides) segment sequences of LAIV strains MM4377M17 and MM4378M18 from Myanmar exhibited 96.4–97.2% nucleotide and 99.0–99.7% amino acid sequence similarities to the prototype LAIV strain BT20 from Guǎngxī, China (Tables S1–S3).

On the other hand, compared to the prototype XSV strain VN1982B4 from *Hipposideros pomona* in Phú Thọ Province [13,17], the nucleotide sequence similarity among the 14 XSV strains (five new strains and nine previously reported strains) amplified from *Hipposideros* bats (Table 3) ranged from 79.2–87.6%, 79.7–86.8% and 77.6–85.8% for the S-, M- and L-genomic segments, respectively (Tables S1–S3). At the deduced amino acid levels, the sequence similarity was considerably higher, ranging from 93.6–99.5% for the nucleocapsid protein, 92.9–97.3% for the envelope glycoproteins, and 91.2–99.5% for the RNA-dependent RNA polymerase (Tables S1–S3).

### 3.3. Phylogenetic Analysis

**Figure 3:**
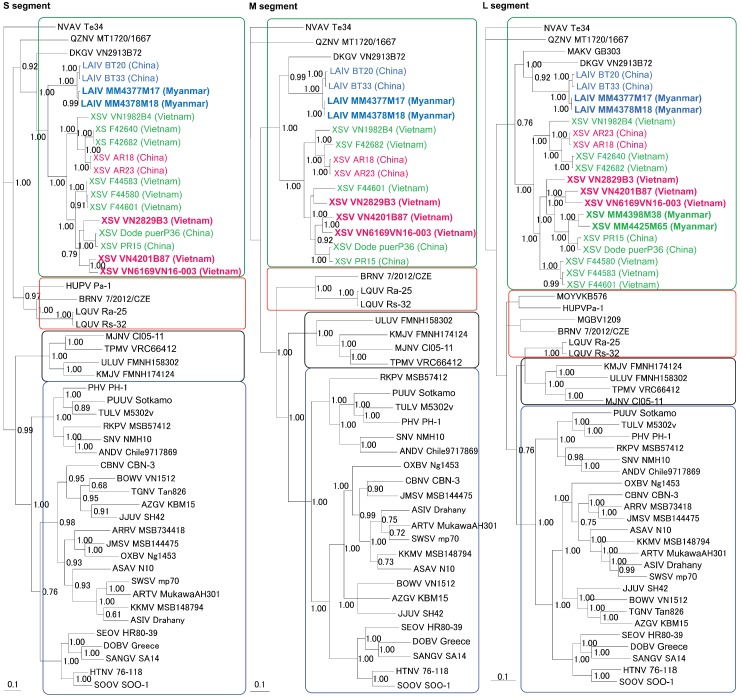
Phylogenetic trees, based sequences of the S-, M-, and L-genomic segments, respectively, generated by the Bayesian Markov chain Monte Carlo estimation method, under the GTR + I + Γ model of evolution. Láibīn virus (LAIV) strains BT20 (S: KM102247; M: KM102248; L: KM102249), BT30 (S: KY662264; M: KY662265; L: KY662266), MM4377M17 (S: MK064114; M: MK064115; L: MK064116), and MM4378M18 (S: MK393932; M: MK393933; L: MK393934) from *Taphozous melanopogon* are shown in blue. Xuân Sơn virus (XSV) strains VN1982B4 (S: KC688335; M: KU976427; L: JX912953), F42640 (S: KF704708; L: KF704713), F42682 (S: KF704709; M: KJ000538; L: KF704714), F44580 (S: KF704710; L: KF704715), F44583 (S: KF704711; L: KF704716), F44601 (S: KF704712; M: KJ000539; L: KF704717), PR15 (S: KY662273; M: KY662274; L: KY662275), Dode puerP36 (S: MG37438; M: MG637437; L: MG637436), MM4398M38 (L: MK393935) and MM4425M65 (L: MK393936) from *Hipposideros pomona* are shown in green. XSV strains VN2829B3 (S: MK393927; M: MK393928; L: LC406451), VN4201B87 (S: MK393929; M: MK393930; L: MK393931), VN6169VN16-003 (S: MK393937; M: MK393938; L: MK393939), AR18 (S: KY662267; M: KY662268; L: KY662269) and AR23 (S: KY662270; M: KY662271; L: KY662272) from *Hipposideros cineraceus* are shown in red. LAIV and XSV strains reported in this study are shown in bold text. Also shown are the phylogenetic positions of other bat-borne hantaviruses, including Dakrong virus (DKGV) strain VN2913B72 (S: MG663536; M: MG663535; L: MG663534) from *Aselliscus stoliczkanus*, Magboi virus (MGBV) strain 1209 (L: JN037851) from *Nycteris hispida*, Mouyassué virus (MOYV) strains KB576 (L: JQ287716) and KB577 (L: KJ000540) from *Neoromicia nanus*, Huángpí virus (HUPV) strain Pa-1 (S: JX473273; L: JX465369) from *Pipistrellus abramus*, Brno virus (BRNV) strains 7/2012 (S: KX845678; M: KX845679; L: KX845680) and 11/2013 (L: KR920360) from *Nyctalus noctula*, Lóngquán virus (LQUV) strains Ra25 (S: JX465415; M: JX465397) from *Rhinolophus affinis*, and LQUV Rs32 (S: JX465422; M: JX465402; L: JX465388) from *Rhinolophus sinicus*, Makokou virus (MAKV) strain GB303 (L: KT316176) from *Hipposideros ruber*, and Quezon virus (QZNV) strain MT1720/1657 (S: KU950713; M: KU950714; L: KU950715) from *Rousettus amplexicaudatus*. Shrew-borne hantaviruses include Cao Bằng virus (CBNV) strain CBN-3 (S: EF543524; M: EF543526; L: EF543525) from *Anourosorex squamipes*, Ash River virus (ARRV) strain MSB734418 (S: EF650086; L: EF619961) from *Sorex cinereus*, Jemez Springs virus (JMSV) strain MSB144475 (S: FJ593499; M: FJ593500; L: FJ593501) from *Sorex monticolus*, Seewis virus (SWSV) strain mp70 (S: EF636024; M: EF636025; L: EF636026) from *Sorex araneus*, Artybash virus (ARTV) strain AH301 (S: KF974360; M: KF974359; L: KF974361) from *Sorex caecutiens*, Kenkeme virus (KKMV) strain MSB148794 (S: GQ306148; M: GQ306149; L: GQ306150) from *Sorex roboratus*, and Asikkala virus (ASIV) strain Drahany (S: KC880342; M: KC880345; L: KC880348) from *Sorex minutus*, as well as Thottapalayam virus (TPMV) strain VRC66412 (S: AY526097; M: NC_010708; L: EU001330) from *Suncus murinus*, Imjin virus (MJNV) strain Cl05-11 (S: EF641804; M: EF641798; L: EF641806) from *Crocidura lasiura*, Azagny virus (AZGV) strain KBM15 (S: JF276226; M: JF276227; L: JF276228) from *Crocidura obscurior*, Tanganya virus (TGNV) strain Tan826 (S: EF050455; L: EF050454) from *Crocidura theresea*, Bowé virus (BOWV) strain VN1512 (S: KC631782; M: KC631783; L: KC631784) from *Crocidura douceti*, Jeju virus (JJUV) strain SH42 (S: HQ663933; M: HQ663934; L: HQ663935) from *Crocidura shantungensis*, Uluguru virus (ULUV) strain FMNH158302 (S: JX193695; M: JX193696; L: JX193697) from *Myosorex geata*, and Kilimanjaro virus (KMJV) strain FMNH174124 (S: JX193698; M: JX193699; L: JX193700) from *Myosorex zinki*. Mole-borne orthohantaviruses include Asama virus (ASAV) strain N10 (S: EU929072; M: EU929075; L: EU929078) from *Urotrichus talpoides*, Oxbow virus (OXBV) strain Ng1453 (S: FJ5339166; M: FJ539167; L: FJ593497) from *Neurotrichus gibbsii*, and Rockport virus (RKPV) strain MSB57412 (S: HM015223; M: HM015222; L: HM015221) from *Scalopus aquaticus*. The single non-bat-borne mobatvirus, Nova virus (NVAV) strain Te34 (S: KR072621; M: KR072622; L: KR072623) from *Talpa europaea*, is also included. Rodent-borne orthohantaviruses include Sin Nombre virus (SNV) strain NMH10 (S: NC_005216; M: NC_005215; L: NC_005217), Andes virus (ANDV) strain Chile9717869 (S: AF291702; M: AF291703; L: AF291704), Prospect Hill virus (PHV) stain PH-1 (S: Z49098; M: X55129; L: EF646763), Tula virus (TULV) strain M5302v (S: NC_005227; M: NC_005228; L: NC_005226), Puumala virus (PUUV) strain Sotkamo (S: NC_005224; M: NC_005223; L: NC_005225), Dobrava virus (DOBV) strain Greece (S: NC_005233; M: NC_005234; L: NC_005235), Hantaan virus (HTNV) strain 76-118 (S: NC_005218; M: NC_005219; L: NC_005222), Soochong virus (SOOV) strain SOO-1 (S: AY675349; M: AY675353; L: DQ056292), Sangassou virus (SANGV) strain SA14 (S: JQ082300; M: JQ082301; L: JQ082302), Tigray virus (TIGV) strain ET2121 (S: KU934010; M: KU934009; L: KU934008), and Seoul virus (SEOV) strain 80-39 (S: NC_005236; M: NC_005237; L: NC_005238). The numbers at each node are Bayesian posterior probabilities (>0.7) based on 150,000 trees: two replicate Markov chain Monte Carlo runs, consisting of six chains of 10 million generations, each sampled every 100 generations with a burn-in of 25,000 (25%). The scale bars indicate nucleotide substitutions per site.

Nearly identical tree topologies, well-supported by posterior node probabilities (>0.70), were generated from an analysis of the S-, M-, and L-segment sequences (Figure 3). Moreover, phylogenetic analyses, using maximum-likelihood and Bayesian methods indicated that LAIV and XSV shared a common ancestry with other mobatviruses (MAKV and DKGV) (Figure 3). The LAIV strains BT20 and BT33 from China, and the LAIV strains MM4377M17 and MM4378M18 from Myanmar formed a monophyletic group, despite the more than 1000-km distance between the trap sites of black bearded tomb bats in China and Myanmar. Nevertheless, LAIV strains from China and Myanmar exhibited geographic clustering, whereas XSV strains from China and Vietnam failed to segregate according to geography or to bat host species.

Also, NVAV from the European mole segregated with the bat-associated mobatviruses, which is reminiscent of trees based on the complete mitochondrial genomes of the European mole and bats [36,37]. The basal position of chiropteran-borne mobatviruses in phylogenetic trees suggests that bats, rather than rodents, may have been the primordial mammalian hosts of ancestral hantaviruses (Figure 3).

**Figure 4:**
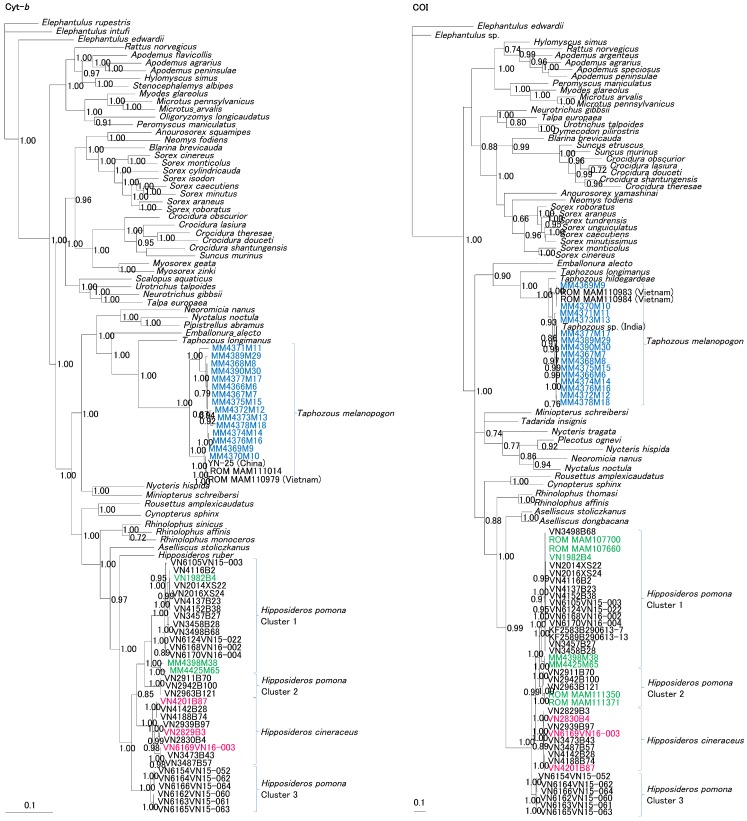
Bayesian phylogenetic trees of the host organisms of mobatviruses reconstructed from the alignments of 1140-nucleotide cytochrome b (Cyt *b*) and 1545-nucleotide cytochrome oxidase I (COI) gene sequences. *Taphozous melanopogon* collected in Myanmar (blue), XSV-positive *Hipposideros pomona* (green) in Myanmar and XSV-positive *Hipposideros cineraceus* (red) in Vietnam are shown. Numbers at the nodes indicate posterior probability values (>0.7) based on 150,000 trees: two replicate Markov chain Monte Carlo runs, consisting of six chains of 10 million generations, each sampled every 100 generations, with a burn-in of 25,000 (25%). Scale bars indicate nucleotide substitutions per site. Gene accession numbers are listed in Table S4.

The molecular identification of LAIV- and XSV-infected bats was confirmed by the amplification and sequencing of the Cyt *b* and COI genes of mitochondrial DNA. By phylogenetic analysis, Pomona roundleaf bats and ashy leaf-nosed bats formed a species complex comprising four closely related clusters, suggestive of local co-circulation in Vietnam (Figure 4).

## 4. Conclusions

Second only to rodents (order Rodentia), bats (order Chiroptera) represent the most species-rich mammalian order, with nearly 1400 species distributed worldwide, except in the frigid polar regions [38,39]. Bats are notorious for hosting many medically important microbial pathogens, and their ability of self-powered flight, longevity and social structures contribute to their role in the transmission of zoonotic diseases [40,41,42].

Formerly divided into the Megachiroptera and Microchiroptera suborders, a new taxonomic nomenclature has been proposed, in which the suborder Yinpterochiroptera comprises megabats or flying foxes (family Pteropodidae), and bats of five microbat families (Craseonycteridae, Hipposideridae, Megadermatidae, Rhinolophidae and Rhinopomatidae), and the suborder Yangochiroptera comprises the remaining microbat families [36].

Thus far, only one hantavirus (QZNV) has been found in a flying fox [16], and four hantaviruses (DKGV, LQUV, MAKV, XSV) have been detected hitherto in bats belonging to the Hipposideridae and Rhinolophidae families [9,13,17,18,19] of the suborder Yinpterochiroptera, and five hantaviruses (BRNV, HUPV, LAIV, MGBV, MOYV) have been detected to date in bats belonging to the Emballonuridae, Nycteridae, and Vespertilionidae families [9,10,11,12,13,14,15] of the suborder Yangochiroptera. Thus, irrespective of the classification, bat species in both suborders have been found to host viruses in the newly created genera of *Loanvirus* and *Mobatvirus*, suggesting that primordial hantaviruses may have emerged in an early common ancestor of bats or other members of the Laurasiatheria superorder, such as shrews and moles [3,12,13,16,20]. A similar conclusion was reached recently from an ancestral state reconstruction analysis of MAKV in the Noack’s roundleaf bat in Gabon [18].

Phylogeographic studies of LAIV and XSV throughout the vast geographic range of the black-bearded tomb bat, Pomona roundleaf bat, and ashy leaf-nosed bat are necessary to obtain additional insights into the biogeographic origin and radiation of mobatviruses and their chiropteran hosts, as well as to clarify whether other bat species harbor LAIV- or XSV-related mobatviruses in Asia.

## Figures and Tables

**Table 1:** Oligonucleotide primers used to amplify mobatvirus RNA from the lung tissues of bats.

Genomic Segment	Primer Name	Sequences (5′ to 3′)	Polarity
S	HTS-3R	TAGTAGTAIGCTCCYT	+
	XSS-147F	CYTWGGRCCTGAACCTGATGA	+
	XSS-467R	GCCTTYARSAGGATRACWACAGG	-
	S-437F	SWGGTCARACTGCHRAYTGG	+
	Cro2R(1126R)	AIGAYTGRTARAAIGAIGAYTTYTT	-
	Cro2F(685F)	AGYCCIGTIATGRGWGTIRTYGG	+
	XSV-S6R	AGITCIGGRTCCATRTCRTCICC	-
	DGS-453R	GTARAAGGRAATGTCASCAGGT	-
	DGS-596F	TGTGTCACTTCCTACTGGTCAG	+
	DGS-704R	GAGCCTTAGTCTCWGCAGCRT	-
	XSS-729R	CCWATIACYCCCATKACWGGRCT	-
	SMGS-1079F	ATIATGGCWTCTAAGCTTGTYGG	+
	XSS-1245F	CTTGGTGATGAYATGGAYTCWGA	+
	XSS-1709R	GCRACTAGTACGTACCTAWAGCGA	-
M	XM-3endR	TAGTAGTAKRCTCCGCARGA	+
	XSM-435R	TTGCCCAGGTCTGCTCAGCA	-
	MKWM-917R	TRTCATGATCTTCICCATRTGG	-
	T-M1199F	TAAVTTCAMCAACATGTCT	+
	T-M1485R	CCAGCCAAARCARAATGT	-
	HTM-1490F	TGTGTICCWGGITTYCATGGIT	+
	XDM-2017R	ACICCRTGWGCTGTRTCYTGCCA	-
	HTM-2409R	CCACAIGCWGTRCAICCWGT	-
	MKWM-2631R	CATGATRTCICCAGGRTCICC	-
	XDM-2841F	TIATGTGGKCTGAYCCWGATGG	+
	XSM-2959R	CTGAACCCCAWGMICCTTCAAT	-
	XDM-3360F	GKWTRTTYCAYGGMAACTGGTGG	+
L	HL-3endR	TAGTAGTAKRCTCCGGA	+
	PHL-173F	GATWAAGCATGAYTGGTCTGA	+
	XAL-948F	CAATMTGAGTATTCMCCWKCTAC	+
	XAL-1534F	CAAARTWYTGGTCTGTYCATGC	+
	XAL-2137F	AGGWGCIAGTGGWGTKTATCC	+
	XSL-2227R	TGCTTCTTCTGTCATWGTICCAYG	-
	HAI-L-F1	ATGTAYGTBAGTGCWGATGC	+
	HAI-L-R1	AACCADTCWGTYCCRTCATC	-
	HAI-L-F2	TGCWGATGCHACIAARTGGTC	+
	HAI-L-R2	GCRTCRTCWGARTGRTGDGCAA	-
	DGL-3225F	TACGTGGIAATTGGTTGCARGG	+
	XSL-3719F	TRGCTGCTKCWCARASTMGKTGTG	+
	XSL-4183R	GTCATAKRCAGGATGCTCWTSTG	-
	XSL-4720F	GATATYAGTGACAGRCARGTTATG	+
	PHL-5167R	CATAYTGYTTHCCTGAATAWGC	-
	HTL-5278F	GTGCAAGSYTAGARATITYYTGGG	+
	XDL-5809R	GCAYTAGGRGGRATWGATGCAGG	-
	XDL-6088R	GTAGRAAATGCTCWATGTCATC	-

Abbreviations: A, Adenine; B, C or G or T; C, cytosine; D, A or G or T; G, guanine; H, or C or T; I, inosine; K, G or T; M, A or C; R, A or G; S, G or C; T, thymine; V, A or C or G; W, A or T; Y, C or T.

**Table 2:** RT-PCR detection of mobatvirus RNA in the lung tissues of bats captured in Myanmar and Vietnam.

Country	Species	Capture Site *	Trap Year	Number Tested	Number Positive †	Mobatvirus Identity §
Myanmar	*Taphozous melanopogon*	Shwe Ba Hill Cave, Sagaing Region	2015	15	2	LAIV
	*Hipposideros pomona*	Nearby forest, Sagaing Region	2015	25	1	XSV
		Nay Pyi Taw Union Territory	2015	1	1	XSV
Vietnam	*Hipposideros cineraceus*	Bắc Hướng Hóa Nature Reserve, Hướng Hóa District, Quảng Trị Province	2013	2	1	XSV
		Xuân Sơn National Park, Tân Sơn District, Phú Thọ Province	2015	3	1	XSV
		Me Linh Station, Phúc Yên District, Vĩnh Phúc Province	2016	1	1	XSV

* Tissues from other bat species captured during the same period were negative for hantavirus RNA by RT-PCR. In Myanmar: 11 greater short-nosed fruit bat (*Cynopterus sphinx*), two Horsfield’s leaf-nosed bat (*Hipposideros larvatus*), one great evening bat (*Ia io*), one painted woolly bat (*Kerivoula picta*), 22 bent-winged bat (*Miniopterus* sp.), five intermediate horseshoe bat (*Rhinolophus affinis*), two woolly horseshoe bat (*Rhinolophus luctus*), 10 Thomas’s horseshoe bat (*Rhinolophus thomasi*), 23 lesser Asiatic yellow bat (*Scotophilus kuhlii*), three long-winged tomb bat (*Taphozous longimanus*). In Vietnam: four Stoliczka’s Asian trident bat (*Aselliscus stoliczkanus*), five greater short-nosed fruit bat (*Cynopterus sphinx*), 13 Horsfield’s leaf-nosed bat (*Hipposideros larvatus*), two Cantor’s roundleaf bat (*Hipposideros galeritus*), 13 Pomona roundleaf bat (*Hipposideros pomona*), one Chinese pipistrelle (*Hypsugo pulveratus*), one black woolly bat (*Kerivoula furva*), one painted bat (*Kerivoula picta*), one long-tongued fruit bat (*Macroglossus sorbinus*), three Western bent-winged bat (*Miniopterus magnater*), four tube-nosed bats (*Murina feae*), two Hutton’s tube-nosed bat (*Murina huttoni*), seven Scully’s tube-nosed bat (*Murina tubinaris*), three Himalayan whiskered bat (*Myotis siligorensis*), one Indochinese mouse-eared bat (*Myotis indochinensis*), three Chinese water myotis (*Myotis laniger*), three wall-roosting mouse-eared bat (*Myotis muricola*), 10 Japanese house bat (*Pipistrellus abramus*), three Indian pipistrelle (*Pipistrellus coromandra*), six Java pipistrelle (*Pipistrellus javanicus*), two least pipistrelle (*Pipistrellus tenuis*), 15 intermediate horseshoe bat (*Rhinolophus affinis*), one Con Dao horseshoe bat (*Rhinolophus chaseni*), 10 least horseshoe bat (*Rhinolophus pusillus*), one Marshall’s horseshoe bat (*Rhinolophus marshalli*), three Indo-Chinese lesser brown horseshoe bat (*Rhinolophus microglobosus*), five Pearson’s horseshoe bat (*Rhinolophus pearsonii*), six Thai horseshoe bat (*Rhinolophus siamensis*), seven Chinese rufous horseshoe bat (*Rhinolophus sinicus*), one lesser brown horseshoe bat (*Rhinolophus stheno*), three Dobson’s horseshoe bat (*Rhinolophus yunanensis*), four lesser Asiatic yellow bat (*Scotophilus kuhlii*), three greater Asiatic yellow bat (*Scotophilus heathii*), and three bamboo bats (*Tylonycteris fulvida*). † RT-PCR amplicons were confirmed as mobatvirus by DNA sequencing. § Mobatvirus: LAIV, Láibīn virus; XSV, Xuân Sơn virus.

**Table 3:** Láibīn virus (LAIV) and Xuân Sơn virus (XSV) in insectivorous bats in Myanmar and Vietnam.

Virus	Strain	Bat Species	Country	Province/Region	S	M	L
LAIV	BT20	*Taphozous melanopogon*	China	Guǎngxī	1935 bp	3908 bp	6531 bp
					KM102247	KM102248	KM102249
	BT33			Guǎngxī	1935 bp	3908 bp	6531 bp
					KY662264	KY662265	KY662266
	MM4377M17 *		Myanmar	Sagaing	1776 bp	3881 bp	6531 bp
					MK064114	MK064115	MK064116
	MM4378M18 *			Sagaing	1798 bp	3707 bp	6531 bp
					MK393932	MK393933	MK393934
XSV	VN1982B4	*Hipposideros pomona*	Vietnam	Phú Thọ	1748 bp	3756 bp	6520 bp
					KC688335	KU976427	JX912953
	F42640			Tuyên Quang	516 bp		567 bp
					KF704708		KF704713
	F42682			Tuyên Quang	1752 bp	663 bp	1160 bp
					KF704709	KJ000538	KF704714
	F44580			Quảng Nam	1728 bp		804 bp
					KF704710		KF704715
	F44583			Quảng Nam	1728 bp		1160 bp
					KF704711		KF704716
	F44601			Quảng Nam	1728 bp	663 bp	1160 bp
					KF704712	KJ000539	KF704717
	PR15		China	Yúnnán	1743 bp	3583 bp	6522 bp
					KY662273	KY662274	KY662275
	Dode puerP36			Shāndōng	1702 bp	2730 bp	4581 bp
					MG37438	MG637437	MG637436
	MM4398M38 *		Myanmar	Sagaing			356 bp
							MK393935
	MM4425M65 *			Nay Pyi Taw			356 bp
							MK393936
XSV	AR18	*Hipposideros cineraceus*	China	Guǎngxī	1752 bp	3753 bp	6521 bp
					KY662267	KY662268	KY662269
	AR23			Guǎngxī	1753 bp	3751 bp	6521 bp
					KY662270	KY662271	KY662272
	VN2829B3 *		Vietnam	Quảng Trị	1660 bp	1754 bp	6521 bp
					MK393927	MK393928	LC406451
	VN4201B87 *			Phú Thọ	1714 bp	3704 bp	6521 bp
					MK393929	MK393930	MK393931
	VN6169VN16-003 *			Vĩnh Phúc	1740 bp	782 bp	3117 bp
					MK393937	MK393938	MK393939

* Mobatvirus strains from this study. bp, base pairs.

## Supplementary Materials

The following are available online at https://www.mdpi.com/1999-4915/11/3/228/s1, Table S1: Nucleotide and amino acid sequence similarities of the S segment of newfound mobatviruses in Myanmar and Vietnam, Table S2: Nucleotide and amino acid sequence similarities of the M segment of newfound mobatviruses in Myanmar and Vietnam, Table S3: Nucleotide and amino acid sequence similarities of the L segment of newfound mobatviruses in Myanmar and Vietnam, Table S4: Gene accession numbers of cytochrome *b* (Cyt *b*) and cytochrome oxidase subunit 1 (COI) sequences.
