# Supplementary material for: Molecular Phylogeny of Mobatviruses (Hantaviridae) in Myanmar and Vietnam
Source: Viruses. 2019 Mar 7;11(3):228. doi: 10.3390/v11030228 (PMC6466252; doi:10.3390/v11030228)
Supplement: Supplementary file 1 [file viruses-11-00228-s001.pdf]

Supplemental Table S1. Nucleotide and amino acid sequence similarities of the S segment of newfound mobatviruses in Myanmar and Vietnam.

S Segment (nt similarity)

Mobatvirus strain	LBNV BT20	LBNV BT33	LBNV MM4377M17	LBNV MM4378M18	XSV VN1982B4	XSV VN4201B87	XSV VN2829B3	XSV N6169VN16-003	XSV F44580	XSV F44583	XSV F44601	XSV F42640	XSV F42682	XSV AR23	XSV AR18	XSV PR15	XSV Dode puerP36
LBNV BT20	–																
LBNV BT33	98.9%	–															
LBNV MM4377M17	96.4%	96.8%	–														
LBNV MM4378M18	97.2%	97.5%	99.2%	–													
XSV VN1982B4	71.0%	71.0%	71.1%	71.2%	–												
XSV VN4201B87	71.1%	71.2%	72.1%	72.0%	80.9%	–											
XSV VN2829B3	70.0%	70.6%	70.5%	70.6%	79.2%	84.1%	–										
XSV VN6169VN16-003	69.6%	69.6%	70.9%	70.8%	80.6%	91.5%	83.9%	–									
XSV F44580	69.9%	70.3%	70.7%	71.1%	80.2%	85.1%	83.4%	83.6%	–								
XSV F44583	70.2%	70.5%	71.0%	71.4%	80.3%	85.4%	83.3%	84.0%	98.8%	–							
XSV F44601	69.9%	70.3%	70.7%	71.1%	80.2%	85.0%	83.3%	83.7%	99.1%	99.3%	–						
XSV F42640	69.6%	69.2%	69.2%	69.4%	85.5%	78.3%	78.9%	77.7%	80.8%	81.0%	81.0%	–					
XSV F42682	71.7%	71.5%	72.1%	72.1%	86.3%	79.4%	79.0%	78.7%	80.8%	81.0%	80.9%	100.0%	–				
XSV AR23	72.1%	72.0%	72.9%	73.1%	87.2%	79.1%	79.0%	79.0%	81.1%	81.2%	81.1%	90.9%	92.1%	–			
XSV AR18	71.9%	71.7%	72.7%	72.8%	87.6%	78.9%	79.2%	78.8%	80.9%	81.0%	80.9%	91.1%	92.1%	99.3%	–		
XSV PR15	71.0%	71.3%	72.0%	72.1%	81.0%	83.8%	84.4%	82.6%	85.5%	85.6%	85.2%	80.0%	81.6%	80.8%	80.9%	–	
XSV Dode puerP36	70.6%	71.0%	71.9%	72.0%	80.8%	84.4%	85.0%	83.3%	85.7%	85.7%	85.3%	80.4%	81.9%	80.7%	80.8%	97.7%	–

NP (amino acid similarity)

Mobatvirus strain	LBNV BT20	LBNV BT33	LBNV MM4377M17	LBNV MM4378M18	XSV VN1982B4	XSV VN4201B87	XSV VN2829B3	XSV VN6169VN16-003	XSV F44580	XSV F44583	XSV F44601	XSV F42640	XSV F42682	XSV AR23	XSV AR18	XSV PR15	XSV Dode puerP36
LBNV BT20	–																
LBNV BT33	100.0%	–															
LBNV MM4377M17	99.0%	99.0%	–														
LBNV MM4378M18	99.7%	99.7%	99.2%	–													
XSV VN1982B4	78.3%	78.3%	78.5%	79.0%	–												
XSV VN4201B87	77.8%	77.8%	78.0%	78.5%	94.9%	–											
XSV VN2829B3	78.1%	78.1%	77.5%	78.1%	93.6%	96.1%	–										
XSV VN6169VN16-003	77.3%	77.3%	77.4%	78.0%	94.6%	99.3%	95.2%	–									
XSV F44580	77.8%	77.8%	78.0%	78.5%	97.0%	96.3%	96.1%	96.0%	–								
XSV F44583	78.0%	78.0%	78.2%	78.7%	97.0%	96.7%	96.5%	96.5%	99.5%	–							
XSV F44601	78.0%	78.0%	78.2%	78.7%	97.2%	96.5%	96.1%	96.3%	99.8%	99.8%	–						
XSV F42640	74.4%	74.4%	73.3%	74.4%	99.4%	93.0%	91.9%	91.9%	95.9%	95.4%	95.9%	–					
XSV F42682	78.3%	78.3%	78.5%	79.0%	99.5%	94.9%	93.6%	94.6%	97.0%	97.0%	97.2%	100.0%	–				
XSV AR23	78.3%	78.3%	78.5%	79.0%	99.5%	94.9%	93.6%	94.6%	97.0%	97.0%	97.2%	100.0%	100.0%	–			
XSV AR18	78.3%	78.3%	78.5%	79.0%	99.5%	94.9%	93.6%	94.6%	97.0%	97.0%	97.2%	100.0%	100.0%	100.0%	–		
XSV PR15	78.3%	78.3%	78.2%	78.7%	96.7%	96.5%	96.5%	96.3%	98.8%	99.3%	99.1%	95.4%	96.7%	96.7%	96.7%	–	
XSV Dode puerP36	78.0%	78.0%	77.9%	78.4%	96.5%	96.3%	96.1%	96.0%	98.6%	99.1%	98.8%	95.3%	96.5%	96.5%	96.5%	99.8%	

Supplemental Table S2. Nucleotide and amino acid sequence similarities of the M segment of newfound mobatviruses in Myanmar and Vietnam.

M Segment (nucleotide similarity)

Mobatvirus strain	LBNV BT20	LBNV BT33	LBNV MM4377M17	LBNV MM4378M18	XSV VN1982B4	XSV VN4201B87	XSV VN2829B3	XSV VN6169VN16-003	XSV F42682	XSV F44601	XSV AR23	XSV AR18	XSV PR15	XSV Dode puerP36
LBNV BT20	–													
LBNV BT33	98.6%	–												
LBNV MM4377M17	96.5%	97.3%	–											
LBNV MM4378M18	96.4%	97.2%	99.9%	–										
XSV VN1982B4	68.1%	68.2%	68.2%	68.3%	–									
XSV VN4201B87	67.7%	67.8%	67.7%	67.8%	79.7%	–								
XSV VN2829B3	70.8%	70.7%	70.8%	70.8%	81.2%	84.3%	–							
XSV VN6169VN16-003	67.7%	68.3%	68.8%	68.9%	80.6%	89.3%	83.6%	–						
XSV F42682	71.2%	71.6%	71.6%	71.8%	86.6%	80.8%	80.5%	83.0%	–					
XSV F44601	71.0%	70.6%	70.1%	70.3%	80.2%	82.4%	83.1%	84.6%	79.6%	–				
XSV AR23	68.7%	68.8%	68.4%	68.5%	86.8%	79.7%	81.5%	80.6%	93.4%	80.5%	–			
XSV AR18	68.6%	68.7%	68.4%	68.6%	86.7%	79.8%	81.5%	80.6%	93.4%	80.5%	99.2%	–		
XSV PR15	68.3%	68.4%	68.6%	68.8%	80.0%	83.9%	85.1%	83.4%	79.6%	83.3%	79.6%	79.5%	–	
XSV Dode puerP36	70.1%	70.1%	70.5%	70.5%	80.6%	84.3%	85.1%	85.0%	80.1%	83.7%	79.9%	79.9%	97.7%	–

GP (amino acid similarity)

Mobatvirus strain	LBNV BT20	LBNV BT33	LBNV MM4377	LBNV MM4378	XSV VN1982B4	XSV VN4201B87	XSV VN2829B3	XSV VN6169VN16-003	XSV F42682	XSV F44601	XSV AR23	XSV AR18	XSV PR15	XSV Dode puerP36
LBNV BT20	–													
LBNV BT33	99.2%	–												
LBNV MM4377	99.0%	99.5%	–											
LBNV MM4378	99.2%	99.6%	99.9%	–										
XSV VN1982B4	71.9%	72.1%	72.2%	72.5%	–									
XSV VN4201B87	71.3%	71.5%	71.9%	72.2%	92.9%	–								
XSV VN2829B3	74.1%	74.3%	74.3%	74.3%	94.4%	95.5%	–							
XSV VN6169VN16-003	70.8%	71.2%	71.2%	71.2%	93.1%	97.3%	92.1%	–						
XSV F42682	73.3%	74.7%	74.7%	74.7%	97.3%	95.5%	94.1%	96.0%	–					
XSV F44601	73.8%	74.2%	74.2%	74.2%	96.8%	96.8%	95.5%	96.0%	96.8%	–				
XSV AR23	71.9%	72.3%	72.4%	72.6%	97.1%	92.9%	93.6%	94.6%	100.0%	96.8%	–			
XSV AR18	71.8%	72.2%	72.3%	72.6%	96.9%	92.8%	93.6%	94.6%	100.0%	96.8%	99.7%	–		
XSV PR15	72.1%	72.3%	72.4%	72.5%	93.7%	95.8%	95.9%	94.6%	95.9%	98.6%	93.5%	93.6%	–	
XSV Dode puerP36	74.1%	74.4%	74.4%	74.4%	94.8%	95.8%	95.5%	95.8%	96.4%	99.1%	94.5%	94.5%	99.3%	–

Supplemental Table S3.

L Segment (nucleotide similarity)

Mobatvirus strains	LAIV BT20	LAIV BT33	LAIV MM4377 M17	LAIV MM4378 M18	XSV VN1982 B4	XSV VN4201 B87	XSV VN2829 B3	XSV VN6169 VN16-003	XSV MM4398 M38	XSV MM4425 M65	XSV F44580	XSV F44583	XSV F44601	XSV F42640	XSV F42682	XSV AR23	XSV AR18	XSV PR15	XSV Dode puerP36
LAIV BT20	–																		
LAIV BT33	98.6%	–																	
LAIV MM4377M17	97.1%	96.9%	–																
LAIV MM4378M18	97.0%	96.9%	99.8%	–															
XSV VN1982B4	72.4%	72.6%	72.4%	72.4%	–														
XSV VN4201B87	72.1%	72.0%	72.4%	72.5%	79.0%	–													
XSV VN2829B3	72.9%	72.7%	72.8%	72.7%	80.6%	84.4%	–												
XSV \VN6169VN16-003	69.9%	69.5%	70.0%	70.0%	78.0%	88.6%	83.2%	–											
XSV MM4398M38	71.9%	71.6%	71.9%	72.2%	77.6%	85.6%	83.7%	82.4%	–										
XSV MM4425M65	72.2%	71.9%	72.2%	71.9%	78.0%	83.2%	82.5%	81.2%	94.1%	–									
XSV F44580	73.6%	73.5%	73.6%	73.7%	81.3%	83.3%	83.0%	81.9%	81.4%	80.3%	–								
XSV F44583	75.4%	75.2%	75.1%	75.2%	81.6%	83.6%	83.7%	82.1%	81.3%	81.3%	97.8%	–							
XSV F44601	75.3%	75.4%	75.2%	75.3%	81.1%	83.1%	83.7%	81.4%	80.6%	80.6%	97.6%	98.5%	–						
XSV F42640	72.0%	72.8%	72.4%	72.6%	83.8%	79.4%	78.9%	79.5%	78.2%	77.2%	80.7%	80.6%	80.7%	–					
XSV F42682	74.9%	74.9%	74.9%	75.0%	85.9%	80.3%	80.1%	79.0%	78.0%	76.8%	80.3%	81.0%	80.9%	99.7%	–				
XSV AR23	72.6%	72.6%	72.6%	72.6%	85.8%	79.5%	80.2%	78.0%	77.6%	78.8%	81.0%	81.7%	81.7%	93.1%	92.6%	–			
XSV AR18	72.4%	72.4%	72.4%	72.4%	85.8%	79.5%	80.1%	77.9%	77.4%	79.1%	80.7%	81.4%	81.3%	92.5%	92.0%	99.3%	–		
XSV PR15	72.4%	72.5%	72.7%	72.7%	79.3%	83.6%	83.4%	82.1%	91.1%	89.9%	83.3%	83.0%	82.8%	79.9%	80.0%	79.5%	79.5%	–	
XSV Dode puerP36	73.9%	74.0%	74.2%	74.2%	80.2%	84.0%	84.1%	82.3%	90.8%	89.1%	82.3%	82.2%	82.0%	79.5%	80.6%	80.0%	80.0%	97.7%	–

LP (amino acid similarity)

Mobatvirus strains	LAIV BT20	LAIV BT33	LAIV MM4377 M17	LAIV MM4378 M18	XSV VN1982 B4	XSV VN4201 B87	XSV VN2829 B3	XSV VN6169 VN16-003	XSV MM4398 M38	XSV MM4425 M65	XSV F44580	XSV F44583	XSV F44601	XSV F42640	XSV F42682	XSV AR23	XSV AR18	XSV PR15	XSV Dode puerP36
LAIV BT20	–																		
LAIV BT33	99.7%	–																	
LAIV MM4377M17	99.4%	99.4%	–																
LAIV MM4378M18	99.4%	99.4%	99.6%	–															
XSV VN1982B4	80.5%	80.5%	80.6%	80.6%	–														
XSV VN4201B87	80.9%	80.8%	80.9%	80.9%	94.1%	–													
XSV VN2829B3	80.6%	80.6%	80.7%	80.7%	93.5%	96.5%	–												
XSV VN6169VN16-003	75.7%	75.4%	75.6%	75.5%	91.2%	98.0%	94.7%	–											
XSV MM4398M38	81.4%	80.5%	81.4%	81.4%	94.9%	96.6%	97.5%	95.6%	–										
XSV MM4425M65	81.4%	80.5%	81.4%	81.4%	94.9%	96.6%	97.5%	95.6%	100.0%	–									
XSV F44580	85.1%	84.3%	85.1%	85.1%	94.0%	97.0%	97.0%	96.9%	97.5%	97.5%	–								
XSV F44583	87.6%	87.1%	87.6%	87.6%	95.9%	97.9%	97.9%	97.2%	97.5%	97.5%	99.6%	–							
XSV F44601	87.8%	87.3%	87.8%	87.8%	96.1%	97.9%	97.9%	97.2%	97.5%	97.5%	99.6%	99.7%	–						
XSV F42640	81.5%	80.4%	81.5%	81.5%	99.5%	95.8%	95.8%	94.9%	94.9%	94.9%	93.7%	93.7%	93.7%	–					
XSV F42682	86.3%	85.8%	86.3%	86.3%	99.5%	96.6%	96.6%	94.9%	94.9%	94.9%	94.0%	95.6%	95.9%	100.0%	–				
XSV AR23	80.8%	80.8%	80.9%	80.9%	97.7%	93.9%	93.4%	91.1%	94.9%	94.9%	94.0%	95.9%	96.1%	100.0%	99.7%	–			
XSV AR18	80.8%	80.8%	80.9%	80.9%	97.6%	93.9%	93.4%	91.2%	94.9%	94.9%	94.0%	95.3%	95.6%	100.0%	99.2%	99.6%	–		
XSV PR15	80.8%	80.8%	80.8%	80.9%	94.6%	97.3%	96.6%	95.4%	100.0%	100.0%	97.0%	98.2%	97.9%	95.8%	96.9%	94.4%	94.4%	–	
XSV Dode puerP36	84.3%	84.2%	84.2%	84.3%	95.9%	97.8%	97.4%	95.8%	99.2%	99.2%	97.4%	98.5%	98.2%	96.3%	97.2%	95.7%	95.7%	99.5%	–

Supplemental Table S4. Gene accession numbers of cytochrome b (Cyt *b*) and cytochrome oxidase subunit 1 (COI) sequences.

Order	Family	Species	Clone	Country	Cyt *b*		COI	
Macroscelidea	Macroscelididae	*Elephantulus edwardii*			DQ901019		NW_006399889	
Macroscelidea	Macroscelididae	*Elephantulus intufi*			DQ901206			
Macroscelidea	Macroscelididae	*Elephantulus rupestris*			DQ901201			
Macroscelidea	Macroscelididae	*Elephantulus sp*					AB096867	
Chiroptera	Emballonuridae	*Emballonura alecto*		Philippines	MK064108		MK410361	*
Chiroptera	Emballonuridae	*Taphozous longimanus*	MM3177B10	Myanmar	KX458068		MK410381	*
Chiroptera	Emballonuridae	*Taphozous melanopogon*	MM4366M6	Myanmar	MK410325	*	MK410404	*
Chiroptera	Emballonuridae	*Taphozous melanopogon*	MM4367M7	Myanmar	MK410326	*	MK410405	*
Chiroptera	Emballonuridae	*Taphozous melanopogon*	MM4368M8	Myanmar	MK410327	*	MK410406	*
Chiroptera	Emballonuridae	*Taphozous melanopogon*	MM4369M9	Myanmar	MK410328	*	MK410407	*
Chiroptera	Emballonuridae	*Taphozous melanopogon*	MM4370M10	Myanmar	MK410329	*	MK410408	*
Chiroptera	Emballonuridae	*Taphozous melanopogon*	MM4371M11	Myanmar	MK410330	*	MK410409	*
Chiroptera	Emballonuridae	*Taphozous melanopogon*	MM4372M12	Myanmar	MK410331	*	MK410410	*
Chiroptera	Emballonuridae	*Taphozous melanopogon*	MM4373M13	Myanmar	MK410332	*	MK410411	*
Chiroptera	Emballonuridae	*Taphozous melanopogon*	MM4374M14	Myanmar	MK410333	*	MK410412	*
Chiroptera	Emballonuridae	*Taphozous melanopogon*	MM4375M15	Myanmar	MK410334	*	MK410413	*
Chiroptera	Emballonuridae	*Taphozous melanopogon*	MM4376M16	Myanmar	MK410335	*	MK410414	*
Chiroptera	Emballonuridae	*Taphozous melanopogon*	MM4377M17	Myanmar	LC406449		MK410415	*
Chiroptera	Emballonuridae	*Taphozous melanopogon*	MM4378M18	Myanmar	MK064111	*	MK410416	*
Chiroptera	Emballonuridae	*Taphozous melanopogon*	MM4389M29	Myanmar	MK410338	*	MK410417	*
Chiroptera	Emballonuridae	*Taphozous melanopogon*	MM4390M30	Myanmar	MK410339	*	MK410418	*
Chiroptera	Emballonuridae	*Taphozous melanopogon*	ROM MAM110983	Vietnam			HM541973	
Chiroptera	Emballonuridae	*Taphozous melanopogon*	ROM MAM110984	Vietnam			HM541972	
Chiroptera	Emballonuridae	*Taphozous melanopogon*	YN-25	China	MG570075			
Chiroptera	Emballonuridae	*Taphozous melanopogon*	ROM MAM111014		EF584220			
Chiroptera	Emballonuridae	*Taphozous melanopogon*	ROM MAM110979	Vietnam	EF584221			
Chiroptera	Emballonuridae	*Taphozous* sp.	CS-2014	India			KM069434	
Chiroptera	Emballonuridae	*Taphozous hildegardeae*		Kenya			JF442692	
Chiroptera	Hipposideridae	*Aselliscus stoliczkanus*		Vietnam	KU161570		LC406447	
Chiroptera	Hipposideridae	*Aselliscus dongbacana*		Vietnam	MG524933	*	LC406434	
Chiroptera	Hipposideridae	*Hipposideros cineraceus*	VN2829B3	Vietnam	LC406452		MK410375	*
Chiroptera	Hipposideridae	*Hipposideros cineraceus*	VN2830B4	Vietnam	LC406453		MK410376	*
Chiroptera	Hipposideridae	*Hipposideros cineraceus*	VN2939B97	Vietnam	LC406454		MK410378	*
Chiroptera	Hipposideridae	*Hipposideros cineraceus*	VN3473B43	Vietnam	LC406455		MK410389	*
Chiroptera	Hipposideridae	*Hipposideros cineraceus*	VN3487B57	Vietnam	LC406456		MK410390	*
Chiroptera	Hipposideridae	*Hipposideros cineraceus*	VN4142B28	Vietnam	MK091935		MK410396	*
Chiroptera	Hipposideridae	*Hipposideros cineraceus*	VN4188B74	Vietnam	MK091936		MK410402	*
Chiroptera	Hipposideridae	*Hipposideros cineraceus*	VN4201B87	Vietnam	KX458067		MK410403	*
Chiroptera	Hipposideridae	*Hipposideros cineraceus*	VN6169VN16-003	Vietnam	MK410352	*	MK410431	*
Chiroptera	Hipposideridae	*Hipposideros pomona* clade 1	VN1982B4	Vietnam	JX912954		MK410367	*
Chiroptera	Hipposideridae	*Hipposideros pomona* clade 1	VN2014XS22	Vietnam	MK091937		MK410369	*
Chiroptera	Hipposideridae	*Hipposideros pomona* clade 1	VN2016XS24	Vietnam	MK091938		MK410370	*
Chiroptera	Hipposideridae	*Hipposideros pomona* clade 1	KF2583B290613-7	Vietnam	MK091939		MK410372	*
Chiroptera	Hipposideridae	*Hipposideros pomona* clade 1	KF2589B290613-13	Vietnam	MK091940		MK410373	*
Chiroptera	Hipposideridae	*Hipposideros pomona* clade 1	VN3457B27	Vietnam	MK091944		MK410387	*
Chiroptera	Hipposideridae	*Hipposideros pomona* clade 1	VN3458B28	Vietnam	MK091945		MK410388	*
Chiroptera	Hipposideridae	*Hipposideros pomona* clade 1	VN3498B68	Vietnam	MK091946		MK410391	*
Chiroptera	Hipposideridae	*Hipposideros pomona* clade 1	VN4116B2	Vietnam	MK091947		MK410394	*
Chiroptera	Hipposideridae	*Hipposideros pomona* clade 1	VN4137B23	Vietnam	MK091948		MK410395	*
Chiroptera	Hipposideridae	*Hipposideros pomona* clade 1	VN4152B38	Vietnam	MK091949		MK410397	*
Chiroptera	Hipposideridae	*Hipposideros pomona* clade 1	MM4398M38	Vietnam	MK064112		MK410419	*
Chiroptera	Hipposideridae	*Hipposideros pomona* clade 1	MM4425M65	Vietnam	MK064113		MK410420	*
Chiroptera	Hipposideridae	*Hipposideros pomona* clade 1	VN6105VN15-003	Vietnam	MK410344	*	MK410424	*

Chiroptera	Hipposideridae	*Hipposideros pomona* clade 1	VN6124VN15-022	Vietnam	MK410345	*	MK410425	*
Chiroptera	Hipposideridae	*Hipposideros pomona* clade 1	VN6170VN16-004	Vietnam	MK410353	*	MK410432	*
Chiroptera	Hipposideridae	*Hipposideros pomona* clade 1	VN6168VN16-002	Vietnam	MK430029	*	MK430032	*
Chiroptera	Hipposideridae	*Hipposideros pomona* clade 1	ROM MAM107660	Vietnam			ABRVN141-06	*
Chiroptera	Hipposideridae	*Hipposideros pomona* clade 1	ROM MAM107700	Vietnam			ABRVN175-06	*
Chiroptera	Hipposideridae	*Hipposideros pomona* clade 2	VN2911B70	Vietnam	MK091941		MK410377	*
Chiroptera	Hipposideridae	*Hipposideros pomona* clade 2	VN2942B100	Vietnam	MK091942		MK410379	*
Chiroptera	Hipposideridae	*Hipposideros pomona* clade 2	VN2963B121	Vietnam	MK091943		MK410380	*
Chiroptera	Hipposideridae	*Hipposideros pomona* clade 2	ROM MAM 111350	Vietnam			ABRVN532-06	*
Chiroptera	Hipposideridae	*Hipposideros pomona* clade 2	ROM MAM 111371	Vietnam			ABRVN549-06	*
Chiroptera	Hipposideridae	*Hipposideros pomona* clade 3	VN6154VN15-052	Vietnam	MK410347	*	MK410426	*
Chiroptera	Hipposideridae	*Hipposideros pomona* clade 3	VN6162VN15-060	Vietnam	MK410348	*	MK410427	*
Chiroptera	Hipposideridae	*Hipposideros pomona* clade 3	VN6163VN15-061	Vietnam	MK410349	*	MK410428	*
Chiroptera	Hipposideridae	*Hipposideros pomona* clade 3	VN6165VN15-063	Vietnam	MK410350	*	MK410429	*
Chiroptera	Hipposideridae	*Hipposideros pomona* clade 3	VN6166VN15-064	Vietnam	MK410351	*	MK410430	*
Chiroptera	Hipposideridae	*Hipposideros pomona* clade 3	VN6164VN15-062	Vietnam	MK430028	*	MK430031	*
Chiroptera	Hipposideridae	*Hipposideros ruber*			EU934474			
Chiroptera	Molossidae	*Tadarida insignis*		Japan	MK410320	*	MK410371	*
Chiroptera	Pteropodidae	*Cynopterus sphinx*		Vietnam	MK064110		MK410368	*
Chiroptera	Pteropodidae	*Rousettus amplexicaudatus*		Philippines	KU950716		MK410365	*
Chiroptera	Rhinolophidae	*Rhinolophus affinis*		China	JX465358		MK410386	*
Chiroptera	Rhinolophidae	*Rhinolophus thomasi*		Myanmar			MK410385	*
Chiroptera	Rhinolophidae	*Rhinolophus sinicus*		China	JX465362			
Chiroptera	Rhinolophidae	*Rhinolophus monoceros*		China	JX465359			
Chiroptera	Vespertilionidae	*Miniopterus schreibersi*		Philippines	MK064109		MK410364	*
Chiroptera	Vespertilionidae	*Plecotus ognevi*		Mongolia			MK410363	*
Chiroptera	Vespertilionidae	*Nyctalus noctula*		Czech Republic	JX570902		KP273590	
Chiroptera	Vespertilionidae	*Neoromicia nanus*		Côte d'Ivoire	EU797428		JF444201	
Chiroptera	Vespertilionidae	*Pipistrellus abramus*		China	JX465352			
Chiroptera	Nycteridae	*Nycteris hispida*			HQ693722		JF442542	
Chiroptera	Nycteridae	*Nycteris tragata*		Malaysia			HM541174	
Eulipotyphla	Soricidae	*Anourosorex yamashinai*		Taiwan			MK410358	*
Eulipotyphla	Soricidae	*Anourosorex squamipe*		Thailand	AB175090			
Eulipotyphla	Soricidae	*Blarina brevicauda*		United States	MK410313	*	MK410357	*
Eulipotyphla	Soricidae	*Chimarrogale platycephala*		Japan			MK410360	*
Eulipotyphla	Soricidae	*Crocidura lasiura*		South Korea	KJ004674		MK410355	*
Eulipotyphla	Soricidae	*Crocidura shantungensis*		South Korea	HQ663932		MK410356	*
Eulipotyphla	Soricidae	*Crocidura obscurior*		Cote d'Ivoire	KC684096			
Eulipotyphla	Soricidae	*Crocidura theresae*		Burkina Faso	DQ521043			
Eulipotyphla	Soricidae	*Crocidura douceti*		Guinea	KC684929			
Eulipotyphla	Soricidae	*Crocidura heresae*		Guinea	DQ521043		JQ732235	
Eulipotyphla	Soricidae	*Sorex caecutiens*		Japan	KF974362		MK410359	*
Eulipotyphla	Soricidae	*Sorex minutissimus*		Japan			MK410421	*
Eulipotyphla	Soricidae	*Sorex roboratus*		Russia	AB175128		KY930906	
Eulipotyphla	Soricidae	*Sorex tundrensis*		Mongolia			MK430030	*
Eulipotyphla	Soricidae	*Sorex cinereus*		United States	FJ667512		JF443848	
Eulipotyphla	Soricidae	*Sorex monticolus*		Canada	FJ667514		JF436792	
Eulipotyphla	Soricidae	*Sorex unguiculatus*		Japan			AB061527	
Eulipotyphla	Soricidae	*Sorex cylindricauda*		Nepal	AB175121			
Eulipotyphla	Soricidae	*Sorex isodon*		China	JX465364			
Eulipotyphla	Soricidae	*Sorex araneus*		Finland	FJ667524		KT210896	
Eulipotyphla	Soricidae	*Sorex minutus*		Czech Republic	GQ494310			
Eulipotyphla	Soricidae	*Suncus murinus*			DQ630386		MK410374	*
Eulipotyphla	Soricidae	*Suncus etruscus*		Iran			MK410384	*
Eulipotyphla	Soricidae	*Neomys fodiens*		Poland	KC537797		GU981249	
Eulipotyphla	Soricidae	*Myosorex geata*		Tanzania	JX193701			

Eulipotyphla	Soricidae	*Myosorex zinki*	Tanzania	JX193702		
Eulipotyphla	Talpidae	*Dymecodon pilirostris*	Japan		MK410422	*
Eulipotyphla	Talpidae	*Urotrichus talpoides*	Japan	EU918371	MK410354	*
Eulipotyphla	Talpidae	*Scalopus aquaticus*	United States	HM461914		
Eulipotyphla	Talpidae	*Talpa europaea*	Hungary	FJ715340	Y19192	
Eulipotyphla	Talpidae	*Neurotrichus gibbsii*	United States	FJ595237	JF435998	
Rodentia	Muridae	*Apodemus argenteus*	Japan		MK410423	*
Rodentia	Muridae	*Apodemus agrarius*	South Korea	AB303225	KY851941	
Rodentia	Muridae	*Apodemus speciosus*	South Korea	AB073811	MK410366	*
Rodentia	Muridae	*Apodemus flavicollis*	Ukraine	AY158451		
Rodentia	Muridae	*Apodemus peninsulae*	South Korea	AB073811	KP671850	
Rodentia	Muridae	*Rattus norvegicus*	China	KY356141	NC_001665	
Rodentia	Muridae	*Hylomyscus simus*	Guinea	DQ212188	JQ667687	
Rodentia	Muridae	*Stenocephalemys albipes*	Ethiopia	AF518346		
Rodentia	Muridae	*Myodes glareolus*		JX477304	KM892824	
Rodentia	Cricetidae	*Microtus pennsylvanicus*	United States	AF119279	KM189812	
Rodentia	Cricetidae	*Microtus arvalis*	Finland	AY220770	KP190310	
Rodentia	Cricetidae	*Oligoryzomys longicaudatus*	Chile	AF346566		
Rodentia	Cricetidae	*Peromyscus maniculatus*	United States	AF119261	MH260579	

* This study
